# Hydrogel Electrolytes for Quasi-Solid Zinc-Based Batteries

**DOI:** 10.3389/fchem.2020.546728

**Published:** 2020-11-04

**Authors:** Kang Lu, Tongtong Jiang, Haibo Hu, Mingzai Wu

**Affiliations:** ^1^School of Physics and Materials Science, Photoelectric Conversion Energy Materials and Devices Key Laboratory of Anhui Province, Anhui University, Hefei, China; ^2^Key Laboratory of Structure and Functional Regulation of Hybrid Materials, Anhui University, Ministry of Education, Hefei, China

**Keywords:** hydrogel, electrolytes, stretchability, self-healing, zinc-based batteries

## Abstract

On account of high energy density depending on the utilized zinc metal anode of high theoretical capacity and its excellent security due to aqueous electrolytes that usually be locked in polymer hosts referred to as hydrogels, quasi-solid zinc-based batteries have been subjected to more and more interest from researchers. The good water retention and electrolyte load capacity of the hydrogel, contributing to the acquirement of high ionic conductivity and durability of the as-obtained quasi-solid electrolyte, play a significant role on the performance of the devices. Moreover, the chemistry of hydrogels can be tuned to endow quasi-solid electrolytes with additional functions in terms of application scenarios of solid-state batteries. Herein, the frontier disciplines of hydrogel electrolytes for Zn-based batteries were reviewed. The cross-linking process of the polymer networks for hydrogel materials with different functions, such as stretchability, compressibility, and self-healing, were also discussed to analyze the properties of the polymer electrolyte. Based on the merits of the functionalized hydrogel, the further application of hydrogel electrolytes in Zn-based batteries is the focus of this paper. The electrochemical performance and mechanical property of Zn-based batteries with functionalized hydrogel electrolytes under extreme conditions were presented to evaluate the crucial role of the polymer hydrogel electrolyte. Finally, the challenges of hydrogel electrolytes for currently developed Zn-based batteries are highlighted with the hope to boost their commercial application in energy conversion devices.

## Introduction

Environmental and energy issues are the main focus of future development for human society. Recently, the rise of flexible/stretchable electronics with the fundamental feature of maintaining normal function under different mechanical deformation such as bending, twisting, stretching, or rolling have promoted increasing demand for compatible power sources capable of conforming to curved surfaces and withstanding equal deformation (Tan et al., 2017). It is well-known that Li-ion batteries (LIBs) are the power supply of traditional silicon-based electronic devices. However, the potential risk caused by leakage of the utilized flammable and explosive organic electrolyte, combined with the escalating prices of scarce lithium resources and cumbersome rigid packaging put a roadblock in the way to the wide application of them in wearable consumer electronics. Thus, the further exploration for efficient, safe, and low-cost batteries is extremely urgent.

Compared with LIBs, by virtue of higher energy density depending on the utilized zinc metal anode of large theoretical capacity (820 mAh g^−1^) and better security endowed by non-combustible aqueous electrolytes, Zn-based batteries stand out as viable candidates (Li et al., 2019; Yang et al., 2019). Environmentally insensitive Zn is a competitive anode alternative to lithium due to its low cost and redox equilibrium potential (Zn/Zn^2+^). There are multiple types of commercial Zn-based batteries (Zhu et al., 2019). The primary Zn-air batteries (ZABs) are characterized by an inherent large theoretical energy density (1,084 Wh kg^−1^), in addition to distinct environmental benignity, and high security (Fu et al., 2015). Zn-ion batteries (ZIB) feature the same high safety and theoretical capacity (Zhang et al., 2016; Wang et al., 2019; Liao et al., 2020). Combined with other characteristic advantages of low cost, abundant resources, and simple fabrication processes, Zn-based batteries have attracted more and more interest, showing broad prospects in the field of future flexible/wearable electronics.

Traditional Zn-based batteries are usually fabricated in undeformable rigid forms, consisting of an anode, aqueous electrolyte solution, and a cathode, encapsulated in rigid packaging. The electrolytes, as the bridge of electrode connection providing an ion conduction channel, directly determines the ionic transport efficiency in the process of the electrochemical reaction and electrochemically stable potential window, which is one of the key factors affecting the electrochemical performance of the devices. The urgently unresolved issues of liquid electrolytes are inevitable evaporation and leakage of solvent water, which further lead to precipitation of hydroxide ions, resulting in an increase of internal resistance and performance degradation. In addition, traditional aqueous electrolytes with liquidity require rough packing, not only increasing the cost, but is also not conducive to wearable applications. Thus, in terms of manufacturing flexible/stretchable Zn-based batteries with both a high deformability and electrochemical performance, more affection factors must be considered.

In the 1990's, solid polymer electrolytes (SPEs), classified as solution-free electrolytes, were made by mixing alkali metal salts into polyethylene oxide (PEO) plasticizers, which efficiently avoid the evaporation and leakage issues of electrolytes, and possess dimensional stability. This makes them a very promising alternative to traditional aqueous electrolytes for flexible/stretchable Zn-based batteries. However, limited by low conductivity (10^−7^-10^−8^ S cm^−1^), SPEs made into Zn-based batteries are not sufficient. Recently, hydrogel electrolytes latched onto aqueous electrolytes in a polymeric matrix became a new method for generating electrolytes for Zn-based batteries. This effectively accelerated the hydroxide-ion conductivity benefiting the improvement of electrocatalytic reaction kinetics compared with SPEs, while alleviating the evaporation and leakage tendency of the solvent more than that of liquid electrolytes, integrating the superiority of liquid electrolytes and SPEs (Xu et al., 2015). In addition, the hydrogel materials can be designed and tuned to achieve special functions by developing polymer engineering technology, which has been seen to greatly enriched the application coverage of the hydrogel family. The formation mechanism of hydrogels with good mechanical properties was attributed to the fact that the polymer gel provides the framework for various functional groups (Gong et al., 2016; Li et al., 2018a). From the perspective of achieving novel hydrogel electrolytes with additional functions, the effects of the polymer gelling agent and the corresponding aqueous electrolyte composition on the properties of hydrogels deserve a higher research effort (Matsuda et al., 2019). Up to now, tremendous effort focused on developing hydrogels with enhanced ionic conductivity, wettability, and mechanical strength for use as quasi-solid electrolytes in Zn-based batteries (Yang et al., 2016). Although the hydrogel can meet the requirements of flexibility as well as possess the advantage of easy handling in the manufacturing process, flexible and smart Zn-based batteries using a hydrogel as an electrolyte is still in its infancy for commercial application. Therefore, further progress in optimizing hydrogel electrolytes, such as selecting the gel agent and the proportion of each introduced element, is expected to provide more insight into the structure and effect relationship of hydrogel electrolytes for developing flexible Zn-based batteries technologies with enhanced battery performance and flexibility.

Herein, recent advancements in the optimization and modification of up-to-date hydrogels and detailed preparation strategy discussions of different works were outlined, followed by the reviewed application of hydrogel electrolytes in Zn-based batteries. Finally, further development directions and challenges of flexible Zn-based batteries were proposed for promoting the application of the new generation of batteries in electric vehicles and commercial portable electronic equipment.

## Hydrogel Materials

On the macro level, hydrogels usually have a wet and soft status endowed by a special structure consisting of crosslinked hydrated polymer chains with mesoporous space, in which the aqueous solution can be trapped. According to previous reports, the amount of trapped solvent water is up to 2,000 times the polymer chains framework weight (Wang Z. et al., 2018). Therefore, hydrogel electrolytes show comparable ionic conductivity to the conventional liquid electrolyte because solutes can diffuse or permeate within the hydrogels, while elastic crosslinked polymer chains maintain the shape and volume under certain conditions, achieving a dimensional stability similar to solid. Thanks to the crosslinking network, hydrogels can absorb and retain a large amount of water. More notably, the absorption of water is closely related to the crosslinking degree. The higher the crosslinking degree is, the lower the water absorption is. Hydrogels can be formed from water-soluble or hydrophilic polymers that contain natural hydrophilic polymer (such as starch, cellulose, alginate, and chitosan) and synthetic hydrophilic polymer (such as acrylic acid (AA) and its derivatives: polyacrylamide (PAM), polyacrylic acid (PAA), polyn-polyacrylamide, and polymethacrylic acid) by chemical or physical crosslinking. The preparation process of hydrogels involves the introduction of some hydrophobic groups and hydrophilic residues in a water-soluble polymer with a reticular crosslinking structure. The hydrophilic residues are combined with water molecules to lock the water molecules in the network, while the hydrophobic residues expand when they touch water. Except for this kind of polymer, the functional additives (such as crosslinking agent) and synthetic environments (e.g., temperature, pH, and ionic strength) are both the main factors that influence the performance of hydrogels. Benefiting from the development of various polymer engineering technologies, many environmentally sensitive hydrogels with salient features, such as variable swelling behavior in response to the change of environment and self-healing ability, were designed and synthesized, which broadened the application prospect of the polymeric hydrogels.

Hydrogel network bonding can be mainly divided into two forms: physical and chemical crosslinking. Physical gels that can be turned into a solution by heating are formed by physical forces, such as electrostatic interaction, hydrogen-bond interaction, and intertwining of chains, which are non-permanent. While chemical gels are three-dimensional network polymers formed by chemical bonding and crosslinking, which are permanent. For example, the H-bonding formed in intermolecular or intramolecular reactions would induce weak physical crosslinking, while covalent bonding with the function of various crosslinking agents would induce strong interactions. The integration of the strong and weak interactions enrich the controllability and diversity of the electrical, mechanical, and biological functions of hydrogels.

The first generation of hydrogels were referred to as conventional single-network (SN) hydrogels, in which catastrophic crack propagation would occur triggered by the rupture of a few polymer strands, resulting in poor mechanical properties, and limiting their modern application. For the sake of preferable hydrogels, of which the shape and strength can be maintained under repetitive mechanical deformation, double-network (DN) hydrogels consisting of a short chain network and a long chain matrix were further proposed and developed. The short chain network, as prestretched polymer strands, exhibit rigid and brittle features, while the long chain network can be referred as coiled strands, which are soft. When the DN hydrogel is stretched, the short-chain matrix first breaks as a sacrificial bond rupture that effectively disperses energy, meanwhile the long-chain network maintains the elasticity and integrity of the hydrogel in the deformation process. In order to achieve the above situation, the designing principles of DN hydrogels have been proposed: (i) a rigid and brittle polymer component contributing to a short chain network and a soft and ductile neutral polymer contributing to a long chain network; (ii) the molar concentration of the long chain network is about 20–30 times of that of the short chain network; (iii) building a strong asymmetric DN structure by a tightly cross-linked network and a loosely cross-linked network (Li et al., 2018a).

Chitosan (CS) belongs to an alkaline polysaccharide with inherent biocompatibility and biodegradability. Through the disposition of alkaline and a monovalent anionic saline solution, a physical microcrystalline matrix and chain-entanglement matrix can form. In terms of low solubility and strong viscosity of long-chain CS, the CS physical network is usually not satisfied by rebuilding hybrid physical-chemical cross-linked DN hydrogels. Yang et al. proposed high-mechanical hybrid DN hydrogels with a simple soaking strategy by integrating short-chain CS with high solubility in neutral water and a covalent PAM network via hydrogen bonding. In such a way, the breakage of CS physical networks can effectively dissipate energy, and thus greatly improve the strength of the obtained DN hydrogels. Based on the aforementioned dual-network crosslinking mechanism, the DN hydrogels show a strong tensile property (Yang et al., 2016).

**Figure 1:**
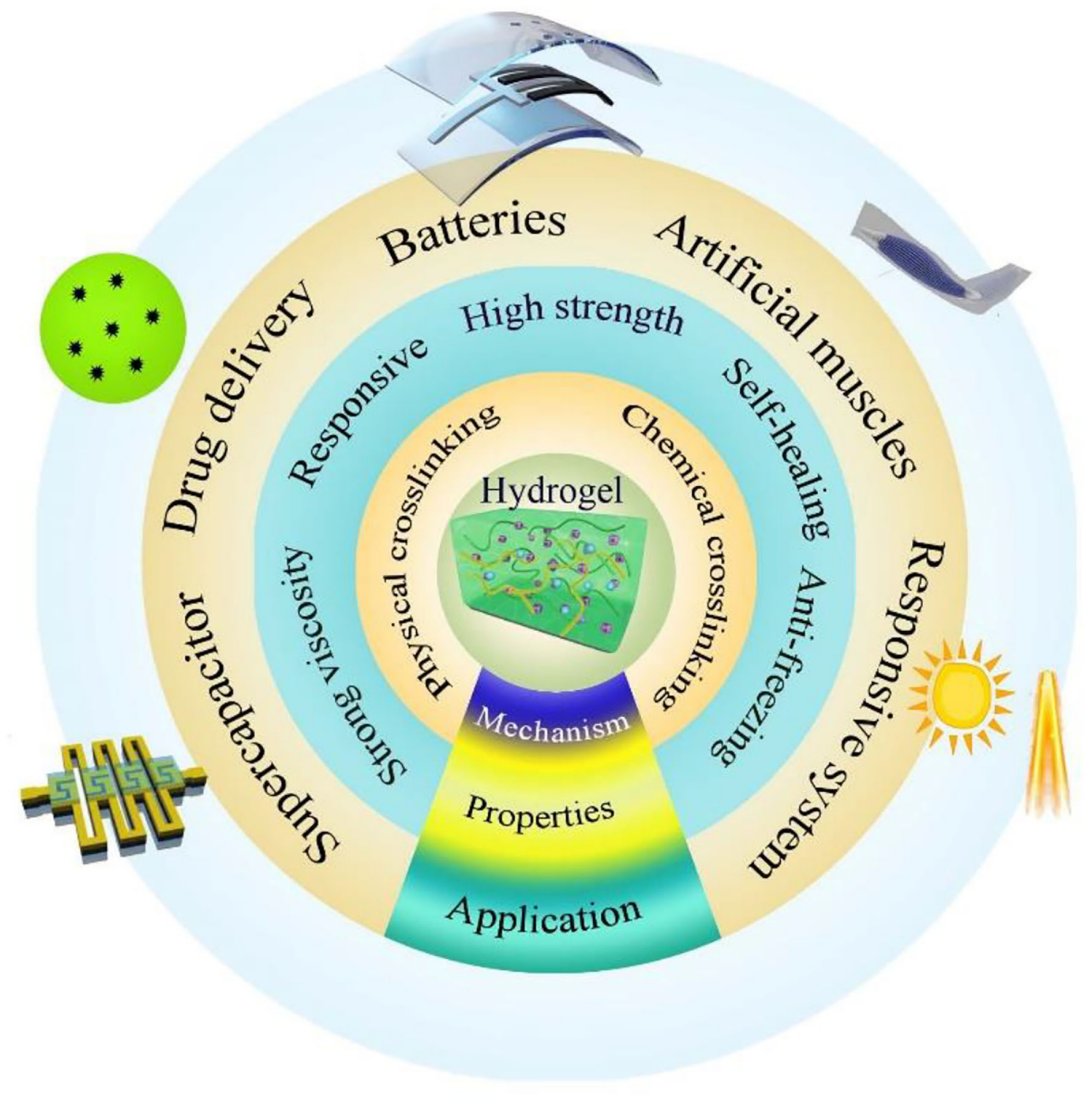
Hydrogel in a wide range of applications.

By virtue of its excellent mechanical properties, unique self-healing properties, and novel anti-freezing feature realized by optimizing a crosslinking strategy, traditional hydrogels have been used in biology and medicine such as rapid sealing, wearable electronic sensors, and healthcare monitoring (Hong et al., 2019). Nevertheless, their electrical properties are often neglected. When salts are dissolved into hydrogels, the dissociated ions can greatly enhance the electrical conductivity of hydrogels. Combined with the stretchable features of the polymer networks and high degree of transparency of water, hydrogels become transparent and stretchable ionic conductors. The hydrophilic polymer networks containing ionic aqueous solutions can form hydrogel electrolytes with large specific surface areas, excellent electron and ion transfer capacity, showing great application potential in deformable energy storage devices for boosting the development of flexible electronics (Figure 1). In the next section, recently reported state-of-the-art hydrogel materials serving as quasi-solid electrolytes in Zn-based batteries (mainly including ZAB and ZIB) will be reviewed.

## Hydrogel Electrolyte for ZN-Based Batteries

The key properties for generic hydrogel electrolytes are ionic conductivity, water-retention capacity, electrolyte permeability, and electrode-electrolyte interaction. The water-retention capacity has significant influence on the stability of the hydrogel electrolyte. The superior ionic conductivity and strong electrode-electrolyte interaction can ensure the smooth progress of reaction during the operation of the batteries. Moreover, the performances of batteries depend on the concentration of electrolytes, so hydrogels with high electrolyte permeability are required. In order to further meet the special requirements of the devices, hydrogel electrolytes are usually endowed with extra functions, such as self-healing and mechanical properties.

Up to date, the reported cathode materials employed in ZIBs mainly include manganese dioxide (MnO_2_), vanadium pentoxide (V_2_O_5_), prussian blue, and conductive polymers. Zn/MnO_2_ batteries, as highly reversible ZIBs, stand out due to their eco-friendliness, low cost, and ease of fabrication. In the process of charge and discharge of ZIBs, the cathode material can be used for the disembedding of Zn^2+^, while the anode will be held responsible for the oxidative dissolution of Zn and reduction of Zn^2+^. The electrolyte for ZIBs is an aqueous solution containing Zn^2+^, which is usually close to neutralization or has weak acidity. Traditional water system electrolytes of ZIBs possess a large amount of water with high reactivity, which will cause electrochemical side reactions and uncontrolled solid-liquid interface reactions. Fortunately, highly efficient hydrogel as transport for cations such as Zn^2+^ and Mn^2+^ not only satisfy the requirements for the controllable shape of the electrolyte but can also provide additional functions for ZIBs applied in portable and wearable electronics.

**Figure 2:**
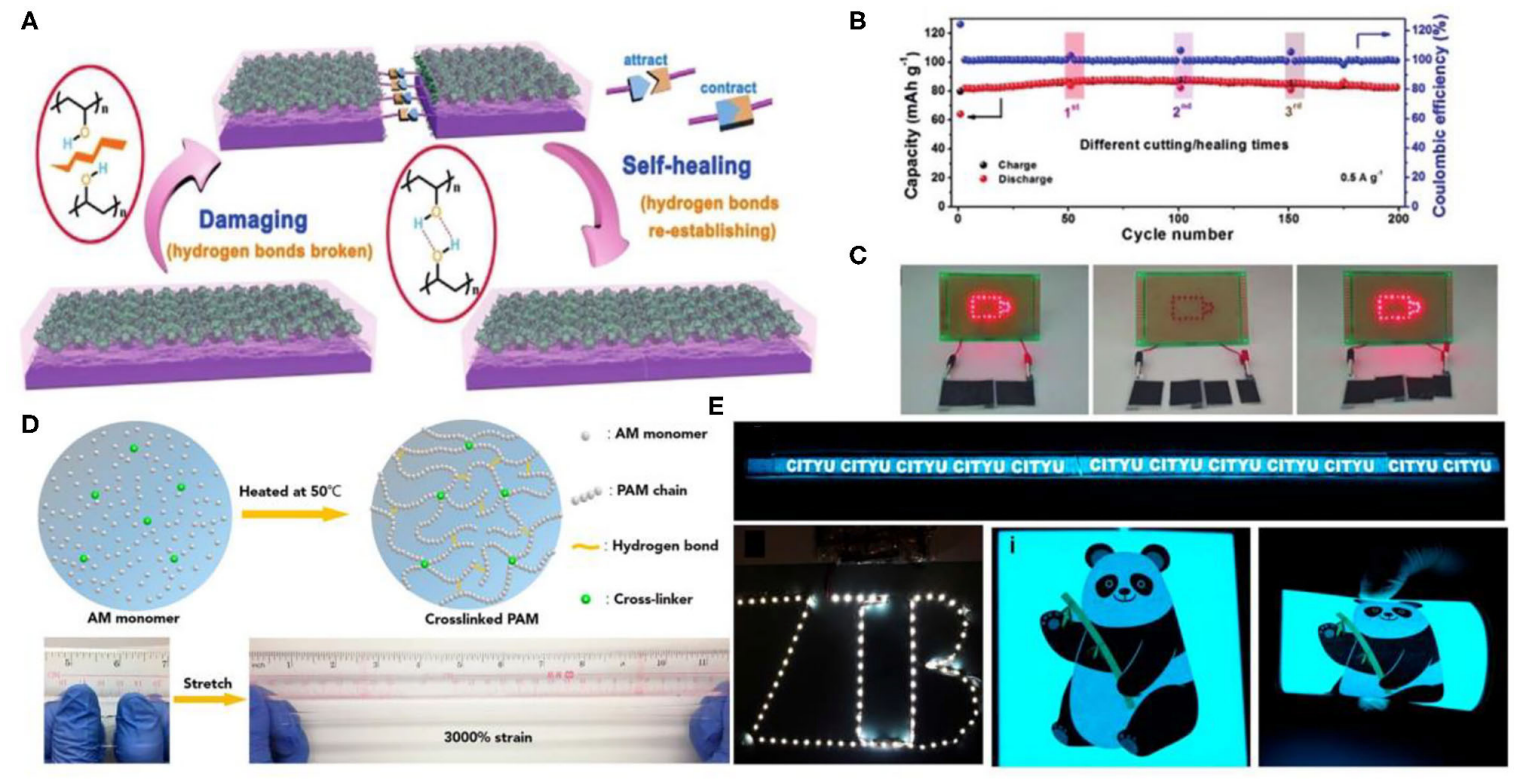
**(A)** Illustration of ZIBs. **(B)** Cycle performance of the self-healing ZIBs at original state and after multiple rounds of cutting/self-healing. **(C)** Photographs of the two self-healing ZIBs in series powering an LED array before cutting (light on), after cutting (light off), and after self-healing (light on again). Reprinted with permission from Huang et al. (2019). Copyright (2019) John Wiley and Sons. **(D)** Schematic diagram and characterization of PAM-based electrolyte. **(E)** Eight yarn batteries were connected in a series to power a 1 m long electroluminescent panel, a long LED belt, and electroluminescent pane under different bending conditions. Reprinted with permission from Li et al. (2018a). Copyright (2018) American Chemical Society.

Poly(vinyl alcohol) (PVA), as a host polymer, has been commonly used in traditional flexible energy storage electronic devices. PVA hydrogels can mix with electrolytic salts, achieving high ionic conductivity (Wang Z. et al., 2018). When suffering from damage, PVA-based hydrogels obtained by a proper strategy would automatically repair. Hydroxy side groups as well as hydrogen bonds contribute to the unique property of PVA. The Huang group prepared a PVA hydrogel electrolyte by a facile freeze/thaw strategy (Huang et al., 2019). The hydrogel prepared with PVA and 2M zinc trifluoromethane-sulfonate [Zn(CF_3_SO_3_)_2_] solution contained less crystalline microdomains. When the PVA chain segments were broken under external force, unconstrained PVA chain segments appeared at the location of the fracture interface. Thus, when broken hydrogels contacted each other again, the unconstrained PVA chain fragments would immediately re-crosslink via hydrogen bonds, as shown in Figure 2A. With the accumulation of time, more PVA chain segments on the fracture interface were re-crosslinked through hydrogen bonds, realizing self-healing without any external stimulus. In addition, PVA/Zn(CF_3_SO_3_)_2_ hydrogel electrolytes also exhibited high ionic conductivity benefiting from their superior 3D porous network. The obtained ZIBs with the PVA/Zn(CF_3_SO_3_)_2_ hydrogel electrolyte can self-heal. Even after undergoing cutting/healing cycles a few times, the electrochemical performance can efficiently restore (Figure 2B). As a demonstration, the LED array can be re-lighted by self-healed batteries (Figure 2C).

Notably, water retention capacity and dissolved salt concentration of the hydrogel electrolyte have significant influence on the performance of ZIBs. However, the concentration of Zn(CF_3_SO_3_)_2_ loaded into the PVA hydrogel can not surpass 3M. As a little gelation area would appear, hydrogels exhibit properties similar to aqueous solution under high concentration (Huang et al., 2019). In addition, the longer carbon chains as well as the lack of hydrophilic groups result in a poor water retention capacity of PVA-based gel electrolytes. Li et al. developed cross-linked PAM (a low-cost polymer containing ketone units) electrolyte (Li et al., 2018b). The as-prepared PAM-based polymer electrolyte possessed 2 M ZnSO_4_ and 0.1 M MnSO_4_, showing good high stretchability up to 3,000% strain (Figure 2D), and high ionic conductivity. Benefiting from the superior capability of the PAM hydrogel, a stretchable yarn-sharped ZIB was fabricated. The device retained 97.2% capacity under 300% strain. Interestingly, the sheared device powered a digital watch. In addition, the battery textile made with the sheared device could light an LED belt (Figure 2E).

Flexible electronic devices often face a variety of external damage and impact in actual use, such as bending, collision, steeping, piercing, and even encounter shearing, fire, water, and other extreme cases. Thus, a high demand is put forward for the anti-destructiveness and reliability of energy storage devices. Hydrogel materials are extremely absorbent, which are usually soft and of low strength. With directional design, hydrogel materials can become very ductile. Li et al. proposed an electrolyte using novel gelatin (a kind of natural biological macromolecule material) and PAM (Li et al., 2018c). The obtained hierarchical polymer electrolyte (HPE) had a liquid-retaining property and salt resistance. Compared with pristine gelatin-based electrolyte (GE), HPE exhibited higher ionic conductivity (1.76 × 10^−2^ S cm^−1^) due to the increased degree of hydrophilicity facilitating ionic transport. In addition, after grafting PAM onto the gelatin chains, the obtained HPE film showed higher strength (7.76 MPa) than that of pristine GE film (1.25 MPa). Benefiting from the great mechanical performance, flexible solid-state ZIB with HPE as the electrolyte were fabricated. More interestingly, the flexible solid-state ZIB could perform normally under various severe conditions. Moreover, Liu et al. prepared a Zn-alginate/PAM dual-crosslinked energy-dissipative hydrogel electrolyte, in which covalently crosslinked PAM were used as the framework, holding the shape of the hydrogel, while alginate chains with Zn^2+^ ions were crosslinked as the second ionic network (Liu et al., 2019a). Once exerting stress, physical bonds from the crosslinked Zn-alginate network would crack to dissipate energy. While upon withdrawing stress, physical bonds would recover, which endowed the Zn-alginate/PAM hydrogel with excellent tensile properties. ZIB with Zn-alginate/PAM hydrogel electrolytes can operate stably under various external mechanical stimuli. Moreover, batteries can be randomly fashioned into anomalous shapes under dramatic deformations and can even withstand the crush of a car.

When the battery is stretched, bent and twisted, shear stresses will inevitably occur. Therefore, it is necessary to design a hydrogel electrolyte to satisfy a higher requirement for shear force tolerance. Wang et al. proposed a nanofibrillated cellulose (NFC)/PAM hydrogel with strong viscosity, which showed remarkably enhanced mechanical properties and enlarged polymer channels contributing to the increased ionic conductivity (Wang D. et al., 2018). The ZIB with NFC/PAM as a hydrogel electrolyte was fabricated combined with sewing techniques. As a demo, the sewed battery as clothes for little toys was used to drive an LED, demonstrating the flexibility and wearability of the obtained battery.

For a long time, in addition to mechanical properties, thermal runaway has been a stumbling block to the progress of smart batteries, which will generate abundant heat during ultra-fast charging-discharging process, or in dangerous conditions such as overcharging and short-circuiting. In order to release the heat accumulated in the battery, the commonly used method at present is to add the physical safety design such as the fuse breaker, extinguishing agent, and closing collecting fluid into the battery. However, these methods only provide one-time protection. Once the temperature cools down, none of these designs would automatically restore the battery to its original operating state. For designing an effective safety switch inside the battery, Mo et al. prepared a smart thermoresponsive polymer electrolyte consisting of proton-incorporated poly(N-isopropylacrylamide-co-Acrylic acid) (PNA) (Mo et al., 2018). The hydrophobic and hydrophilic segments in PNA facilitated reversible sol-gel transition. Carbonyl/imide groups contributed to hydrophilic force. Moreover, isopropyl groups contributed to hydrophobic force. At a low temperature situation, the effect of hydrophilic force played dominant roles, inducing that the electrolyte presented solution properties. However, at a higher temperature, the effect of the hydrophobic force became apparent inducing the appearance of gels. When the temperature decreased again, hydrogen bonds were rebuilt. Therefore, with the changing of temperature, the reversible sol-gel transition process would appear so that the resistance of the PNA electrolyte changed correspondingly. As an example, a PNA sol-gel electrolyte was used to fabricate a ZIB battery. When the temperature increased, the specific capacity obviously dropped. The fabricated rechargeable battery exhibited a self-protection behavior. The LED bulbs can be powered by Zn/α-MnO_2_ batteries. Upon being heated to 50°C, the batteries exhibited a significant reduction in power output until the LED was dimmed out after 30 s, which demonstrated that it is feasible to design smart batteries with thermal self-protection behavior based on the temperature-responsive dynamic electrochemical performance of electrolytes.

**Figure 3:**
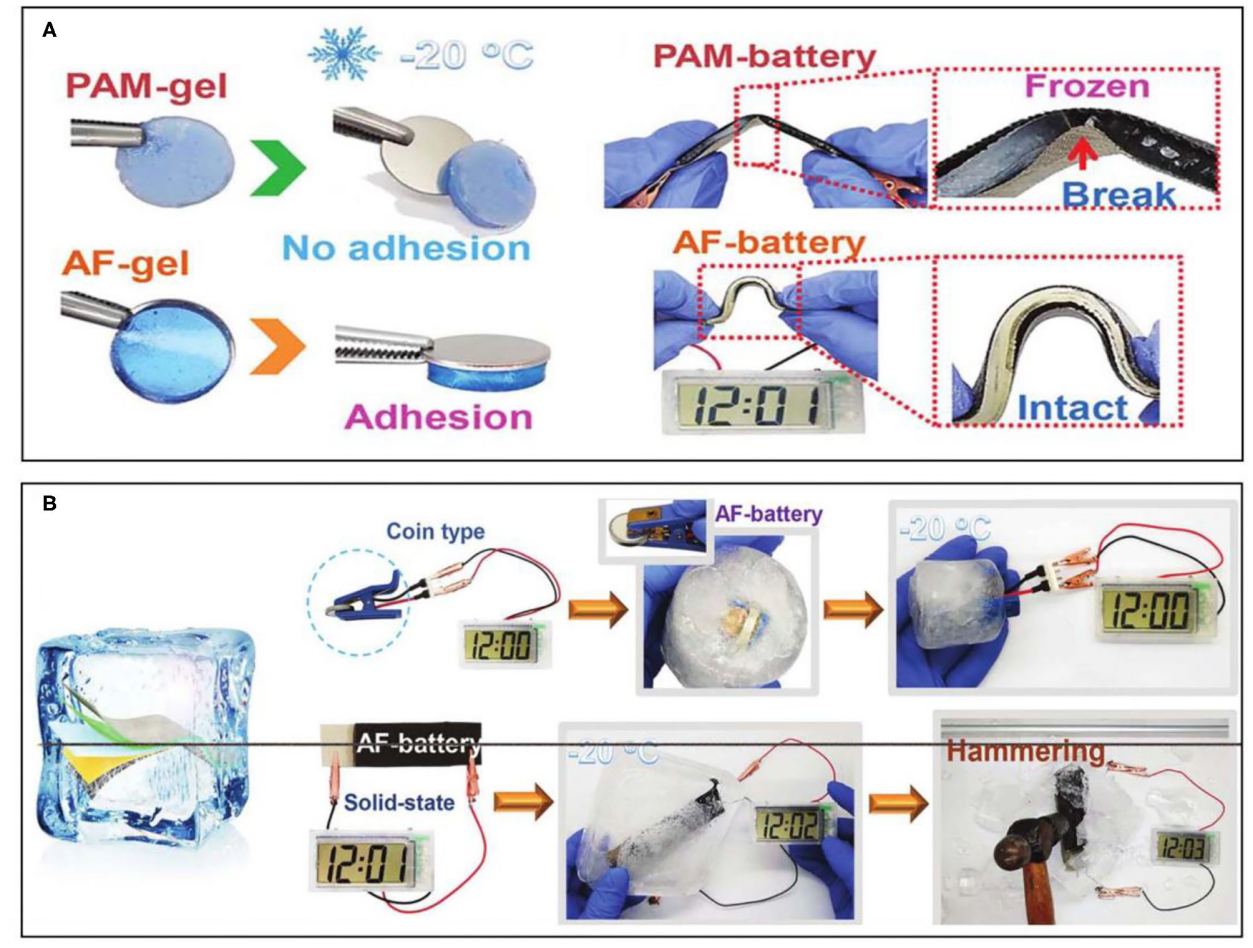
**(A)** Comparisons of the adhesion force and bending demonstration of PAM-battery and AF-battery at −20°C. **(B)** Application demonstration of the AF-battery at subzero temperatures. Reprinted with permission from Mo et al. (2019). Copyright (2019) Royal Society of Chemistry.

Apart from a thermoresponsive property, an anti-freezing featured hydrogel can be prepared by polymer design. At subzero temperatures, devices consisting of hydrogel electrolytes are difficult to operate normally, not to mention maintaining their flexibility due to the freezing of the hydrogel electrolyte. In order to broaden the battery application scenarios, it is necessary to design ideal anti-freezing hydrogel electrolytes with a lower freezing point. One method of realizing an anti-freezing feature is to add lipophilic components to the hydrogel network, but it requires more complex synthesis steps. Therefore, many researchers are usually devoted to the introduction of a high concentration of solute (e.g., glycol, glycerin) into the hydrogel to reduce the freezing point of the water. Based on this strategy, Mo et al. introduced ethylene glycol that as a low molecular vicinal alcohol would usually be used as non-toxic inhibitors for water freezing and designed an anti-freezing (AF) hydrogel electrolyte (Mo et al., 2019). The involved preparation process and design of the EG-waPUA/PAM-based dual crosslinked hydrogel were relatively successful by copolymerizing. Similar to the two-network hydrogel mentioned above, the EG-waPUA polymer chains mainly played a role in strengthening the network matrix, while the PAM polymer chains were used to dissipate energy under deformation. Therefore, the dual crosslinked network can be dynamically recombined under deformation. The obtained AF-gel can not only maintain high adhesiveness, but can also exhibit superior mechanical properties without damage under bending at −20°C (Figure 3A). The further fabricated freeze-resistant flexible ZIB can deliver high capacity retention even if in an extremely cold temperature, indicating the anti-freezing performance of the battery. A coin-type AF-battery in a frozen environment can still power an electronic watch. Moreover, the integrated AF-batteries can power an electronic watch, even sealed in solid ice (Figure 3B), which exemplify the feasibility of wearable applications of the obtained AF-batteries in extremely cold conditions.

**Figure 4:**
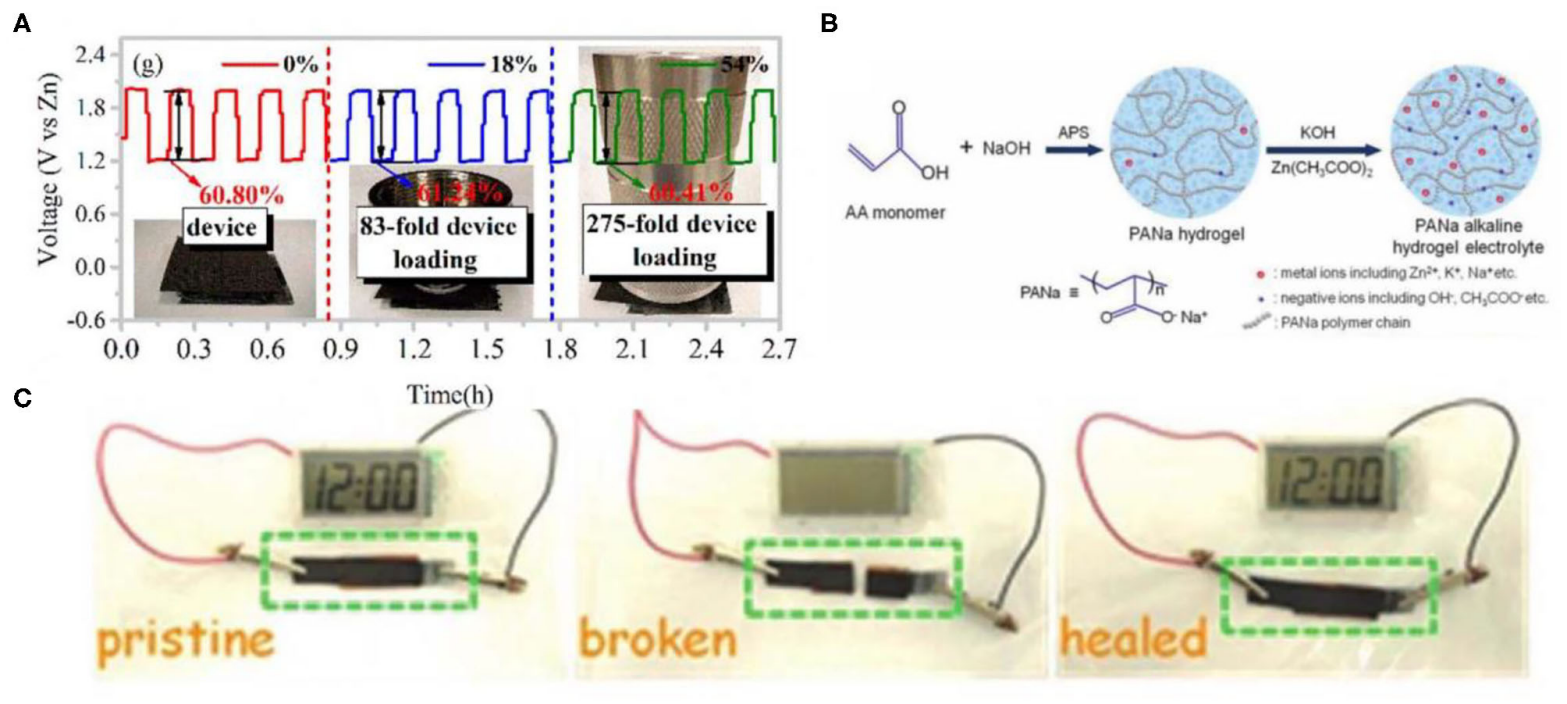
**(A)** Galvanostatic discharge-charge cycling curves at a current density of 5 mA cm^−2^. Reprinted with permission from Ma et al. (2018a). Copyright (2018) American Chemical Society. **(B)** Synthesis of the PANa hydrogel electrolyte. Reprinted with permission from Huang et al. (2018a). Copyright (2018) John Wiley and Sons. **(C)** Photographs of the self-healing aqueous battery powering a clock before cutting (left), after cutting (middle), and after self-healing (right). Reprinted with permission from Huang et al. (2018b). Copyright (2018) John Wiley and Sons.

For ZAB, in the discharge process, Zn is oxidized, releasing electrons. Meanwhile, oxygen molecules in cathode electrode were reduced. The generated hydroxide ions in the oxygen reduction process further migrate to the anode electrode and transform into ${\text{Zn}(\text{OH})}_{4}^{2-}$. When the supersaturate entered the electrolyte, ${\text{Zn}(\text{OH})}_{4}^{2-}$ would be transformed into ZnO. Zn//NiCo batteries have a similar structure to ZABs, in which the NiOOH/CoOOH cathode instead of the air electrode take part in the redox reaction (Li et al., 2019). Different from ZIBs, the electrolytes of ZABs and Zn//NiCo batteries usually adopt alkaline electrolytes. For achieving high power density, strong alkaline electrolytes containing 6 M KOH are usually chosen. However, traditional PVA gels possess low KOH-holding capacity as well as a weak water retention capacity. So, the conventional PVA/KOH hydrogel electrolyte usually exhibited low ion-conductivity. Therefore, it is urgent to construct alkali-resistant electrolytes with a high ion transfer rate, excellent water retention, and strong interaction between the electrode and electrolyte during charge and discharge, as well as exhibiting decent mechanical properties. Ma et al. reported a compressible PAM hydrogel electrolyte (Ma et al., 2018a). In this electrolyte, reversible hydrogen bonds endowed by PAM chains had a weaker force, which ensured that the PAM chains could be dynamically broken and recombined for dissipating the applied energy, contributing to superior mechanical properties. The ZAB assembled with the PAM alkaline hydrogel could maintain their electrochemical performance in the case of compression (Figure 4A). After 500 repetitive compressions, the electrical properties of the battery hardly diminished. Obviously, compressible PAM hydrogel electrolytes laid the foundation for compressible ZABs with good mechanical capability.

Generally, hydrogels constantly absorb or lose water in the air as the ambient humidity changes, resulting in a fluctuation of the performance of the hydrogel. Dissolving the hydrating salt in the hydrogel to form hydrated ions can effectively reduce the vapor pressure of the water and thus slow/inhibit evaporation. Many polymer hydrogels show excellent mechanical properties. However, when incorporated with strong alkaline electrolytes, the mechanical robustness of them would be broken (Huang et al., 2017; Ma et al., 2018b). Therefore, other types of versatile hydrogels that are highly stable in strong alkaline corrosive media still need to be further explored. Huang et al. reported a new electrolyte comprising of Zn(CH_3_COO)_2_, (0.2 M)/KOH (6 M), and sodium poly-acrylate hydrogel (PANa) that is highly stable in strong alkaline corrosive media and a well-known superabsorbent polymer, which can keep excellent mechanical performance (Huang et al., 2018a). A PANa hydrogel was synthesized through the polymerization of AA monomers disposed with NaOH (Figure 4B). Based on the superior stability performance of the PANa hydrogel, the solid-state PANa-based Zn//NiCo battery and ZAB afforded higher cycling stability and high capacities. In addition, Tang et al. introduced a poly-acrylamide-co-polyacrylic acid (PAM-co-PAA) gel electrolyte for fabricating ZABs (Tang et al., 2020). Thanks to abundant acylamino/carboxyl, the PAM-co-PAA hydrogel showed superior moisture retention under repeated tensile deformation. The as-prepared PAM-co-PAA copolymer can still retain more than half the water in the atmosphere for 10 days. The integrated ZABs with the PAM-co-PAA/KOH electrolyte in series interconnection were wearable, displaying excellent flexibility. In addition, they were powerful enough to charge a smartphone.

The stretchability and compressibility of energy devices are also important for their performance as wearable electronics. However, up to date, few efforts have been devoted to the design and fabrication of batteries with intrinsic stretchability and compressibility due to the lack of electrolytes and electrodes with a stretchable and compressible property. Liu et al. reported that the PANa hydrogel exhibited superior stretchability and high compressibility. The newly developed battery with the PANa hydrogel as an electrolyte can be efficiently stretched and compressed while maintaining good capacity (Liu et al., 2019b). It can be concluded from the above discussion that the PANa hydrogel not only possesses a fast ion transfer property, strong water-locking ability, but also exhibits intrinsic excellent tensile and compressibility.

The emerging DN hydrogels have gained popularity due to their superior mechanical properties. The reported cross-linking modes of DN hydrogels have three species: chemistry, physics, and hybridization. The physical interactions for hydrogels have many types such as hydrogen bonding and crystalline domains (Gong et al., 2016). Compared with the hydrogels based on chemically cross-linking, physically cross-linked hydrogels are characterized by dynamical performance. Therefore, the construction of physically cross-linking within a hydrogel matrix is the key for achieving stretchability and self-healing ability. However, mechanical ability endowed by one physically cross-linked network is still limited, hindering their application in energy storage devices with desirable multi-function. Constructing the multiple physically cross-linked matrix has become a popular way to design multi-functional hydrogel electrolytes. Ma et al. designed an alkaline-resisting dual-network hydrogel electrolyte consisting of PANa chains, cellulose (potassium hydroxide stabilizer), and cross-linkers N, N″-methylenebis acrylamide (MBAA) (Ma et al., 2019). The interactions of the PANa and MBAA/cellulose chain promoted covalent cross-linking. In addition, PANa and cellulose contributed to hydrogen bonds/chain entanglements. In this hydrogel electrolytes, multiple forces synergistically strengthened the mechanical robustness and stretchability of the hydrogel. Thanks to the dual-network formed by the physical and chemical cross-linking reaction, the alkaline hydrogel electrolyte possessed over 1,000% stretchability, and good flexibility. Surprisingly, with the usage of the alkali-resistance PANa-cellulous hydrogel, the designed flat-structured and fiber-shaped device showed a super-stretchable performance. The obtained device offered stable power output to power an electronic watch under tensile deformation. The obtained fiber ZAB disposed by hydrophobic treating can power an electronic watch in a water environment. As a result, the developed alkali-resisting and stretchable electrolyte boosted the development of waterproof wearable ZABs.

The hydrogel materials with self-healing ability can automatically repair damage and restore their structure and function, thus improving the safety, reliability, and durability and further extending their service life. The hydrogel network with high strength needs high-intensive and stable crosslinking polymerization, while the hydrogel with self-healing needs dynamic reversible crosslinking. Therefore, high strength and self-healing in essence is contradictory. So the preparation of a hydrogel that possesses simultaneously high mechanical strength and excellent self-healing properties is an important issue in the field of hydrogels (Shao et al., 2018). Moreover, the alkaline hydrogel electrolyte usually displays more unavoidable problems (such as low coulombic efficiency, capacity attenuation) than that of neutral cases (Huang et al., 2019), which creates issues when designing self-healing alkaline hydrogel electrolytes. PANa has high compatibility with abundantly unconstrained Zn^2+^ and OH^−^ ions that is conducive to ion transportation, and thus is usually used as the hydrogel electrolyte matrix. Non-covalent crosslinking or dynamic covalent bonding can endow hydrogels with additional self-healing properties. Huang et al. synthesized a self-healable hydrogel electrolyte consisting of PANa, ferric ions (Fe^3+^), 0.2 M Zn(CH_3_COO)_2_, and 6 M KOH (Huang et al., 2018b). Among them, Fe^3+^ were used as crosslinkers to strengthen the self-healing ability of the hydrogels. Fe^3+^ ions promoted the interactions of polymer chains forming physical cross-linking, which determined the self-healing effect. As a result, the healed PANa-Fe^3+^ hydrogel exhibited higher tensile strain (around 1,000%) and strength (205 kPa) than those of the pure PANa. When the obtained PANa-Fe^3+^ hydrogel was broken and the broken parts were contacted again, the integral hydrogel network would reconstruct due to the reformation of ionic bonds through connecting Fe^3+^ with the acrylate groups. Due to the higher ionic conductivity and better self-healing property of the PANa-Fe^3+^ hydrogel, the fabricated NiCo||Zn batteries with a PANa-Fe^3+^ hydrogel electrolyte still delivered high self-healing efficiency (over 87%) after undergoing four cycles of repeated cutting and healing processes. As a demonstration, the clock powered by a self-healing NiCo||Zn battery would turn off immediately once the battery was completely cut through and would turn on again when the broken parts had direct contact with each other (Figure 4C), which demonstrated that self-healing alkaline PANa-Fe^3+^ hydrogel electrolytes could lay the foundation of intrinsic self-healing batteries. From the above discussion, it can be deduced that polyelectrolytes should meet two fundamental conditions to acquire self-healing properties: (i) being compatible with ions to achieve high ionic conductivity, (ii) containing reversible non-covalent bonds (such as hydrogen bonds, ionic bonding) for rebuilding the broken network.

For Zn-based batteries, the reversibility of Zn deposition also has a relationship with the electrolyte, which greatly affects the rechargeability of devices. In traditional Zn-based batteries with an aqueous electrolyte, water with high reactivity is abundant, which induces the formation of Zn dendrite and by-products. However, low active water content in hydrogel electrolytes can alleviate these problems to some extent. For example, the acrylate groups in sodium polyacrylate hydrogel (PANa) electrolytes have a negative charge and thus generate electrostatic action with positive Zn ions. On account of this interaction, the quasi-solid electrolyte interface formed in the surface of electrode can effectively suppress the formation of Zn dendrites (Huang et al., 2018a).

This review mainly focused on the hydrogel electrolyte performance and special function of the fabricated batteries. As shown in Table 1, these batteries fabricated with hydrogel electrolytes not only provide special functions, but also deliver excellent electrochemical performance, exhibiting broad prospects as a micro power source for flexible electronic devices.

**Table 1:** Comparison of functional zinc-based battery.

**Type of battery**	**Hydrogel electrolyte**	**Electrolyte performance**	**The physical and electro-performance of battery**	**References**
Zn-MnO_2_ battery	PAM-based electrolyte containing ZnSO_4_ (2 M) and MnSO_4_ (0.1 M)	Tensile strength: 273 kPa, stretchability: 3,000% strain, ionic conductivity: 17.3 × 10^−3^ S cm^−1^	Specific capacity: 302.1 mAh g^−1^, energy density: 53.8 mWh cm^−3^, 98.5% capacity retention after 500 cycles	Li et al., 2018b
Zn-MnO_2_ battery	Gelatin-g-PAM electrolyte containing ZnSO_4_ (2 M) and MnSO_4_ (0.1 M)	Ionic conductivity: 1.76 × 10^−2^ S cm^−1^, strength: 7.76 MPa	Areal energy density: 6.18 mWh cm^−2^, power density: 148.2 mW cm^−2^, specific capacity: 306 mAh g^−1^	Li et al., 2018c
Zn-MnO_2_ battery	Zn-alginate/PAM electrolyte containing ZnSO_4_ (2 M) and MnSO_4_ (0.1 M)	Tensile strength: 51.83 kPa, stretchability: 500% strain, ionic conductivity: 43.2 mS cm^−2^	Specific capacity: 300.4 mAh g^−1^ at 0.11 A g^−1^, 82% capacity retention (500 cycles at 0.88 A g^−1^)	Liu et al., 2019a
Zn-MnO_2_ battery	NFC/PAM electrolyte containing ZnSO_4_ (2 M) and MnSO_4_ (0.2 M)	Ionic conductivity: 22.8 mS cm^−1^, stretchability: 1,400% strain	Specific capacity: ~200 mAh g^−1^ at 4 C, 88.3% capacity retention after 1,000 cycles at 4 C	Wang D. et al., 2018
Zn -MnO_2_ battery	PNA sol-gel electrolyte containing ZnSO_4_ (0.3 M) and MnSO_4_ (0.015 M)	Resistance: 18.1 MΩ at room temperature; 160.9 MΩ at 70^o^C	Specific capacity: 145 mAh g^−1^ at the current density of 0.1 A g^−1^	Mo et al., 2018
Zn-MnO_2_ battery	EG-waPUA/PAM based electrolyte containing ZnSO_4_ (2 M) and MnSO_4_ (0.1 M)	Ionic conductivity: 16.8 mS cm^−1^, ionic conductivity: 14.6 mS cm^−1^ at −20°C	Specific capacity: 275 mAh g^−1^, current density: 0.2 A g^−1^, volumetric energy density: 32.68 mWh cm^−3^ at normal temperature	Mo et al., 2019
Zinc-air battery	PAM electrolyte containing Zn(CH_3_COO)_2_ (0.2 M) and KOH (6 M)	–	Charge-discharge voltage gap: 0.78 V at 5 mA cm^−2^, power density: 118 mW cm^−2^	Ma et al., 2018a
Zn//NiCo and Zn-air batteries	PANa electrolyte containing Zn(CH_3_COO)_2_ (0.2 M) and KOH (6 M)	Ionic conductivity: 0.17 S cm^−1^	Capacities: ~260 mAh g^−1^_NiCohydroxide_ and ~800 mAh g^−1^_Zn_, cycling stability: 65% retention after 16,000 cycles for Zn//NiCo battery; 60 h (800 cycles at 2 mA cm^−2^) for Zn-air battery	Huang et al., 2018a
Zn-air battery	PAM-co-PAA electrolyte containing Zn(CH_3_COO)_2_ (0.2 M) and KOH (6 M)	Stretchability: 2,700% strain, tensile strength: 102.8 KPa, water retention: 68.7% in the air for 10 days	Specific capacity: 738 mAh g^−1^, cycling stability: 35 h, energy densities: 7.53 mWh cm^−2^/900.4 Wh kg^−1^	Tang et al., 2020
NiCo//Zn battery	PANa electrolyte containing Zn(CH_3_COO)_2_ (0.2 M) and KOH (6 M)	Stretchability: up to 1,700% strain, compressibility: 80%	Specific capacity: 71.8 mAh g^−1^, stretchability: 400% strain, compressibility: 50% strain	Liu et al., 2019b
Zi-air battery	PANa cross-linked by cellulose chains and MBAA anchors electrolyte containing Zn(CH_3_COO)_2_ (0.2 M) and KOH (6 M)	Ion conductivity: 0.28 S cm^−1^	Power density: 108.6 mW cm^−2^	Ma et al., 2019
NiCo||Zn battery	PANa-Fe^3+^ electrolyte containing Zn(CH_3_COO)_2_ (0.2 M) and KOH (6 M)	Stretchability: ~1,000% strain, tensile strength: 205 kPa	Specific capacity: ~250 mAh g^−1^	Huang et al., 2018b

## Conclusion and Perspective

In this article, the recent advances of Zn-based batteries with rubbery stretchability, self-healing ability, and additional smart functions using hydrogels as electrolyte materials were reviewed. The electrode active materials and electrolytes are the key components that determine the performance of devices. The superior catalysts in air-electrodes ensure excellent redox performances. While the electrolyte materials determine the ionic conductivity for electrochemical reactions and act as a bridge connecting the cathode and the anode, which also have an intimate relationship with power density. In addition, the stretchability, self-healing ability, and toughness of electrolyte materials contribute to the smart function of the whole Zn-based battery.

To date, various endeavors have been devoted to exploring electrode active materials, but advances on superior hydrogel electrolytes for Zn-based batteries is relatively lacking and limited. Hydrogel electrolytes involve ionic conductivity, electrode/electrolyte interfaces, stretchability, and flexibility which enrich the research dimensions of the batteries field. When encountering mechanical deformations, the electrolyte and electrode materials are inclined to cause dislocation. Conventional liquid electrolytes combined with separator configuration easily detach from the electrode. In addition, a separator that is usually inactive can not deliver additional intelligent functions. Hydrogels with strong water retention capacity and customizable dimensional shape are ideal carriers for a variety of electrolytes. The obtained hydrogel electrolytes can not only integrate liquid electrolytes and separator functions into one, but can also improve the interfacial adhesion to electrode material and provide additional intelligent functions. Although hydrogel electrolytes applied in Zn-based batteries have many advantages, there are still some problems that urgently need to be solved. In order to satisfy the application situation of flexible and wearable Zn-based batteries, it is still necessary to address the following problems related to hydrogel electrolytes in the future exploration for Zn-based devices with mechanical flexibility and intelligent function.

1. Exploring hydrogel electrolytes with good water retention. The energy density, power density, and cycle life of batteries have a close relationship with the durability of the hydrogel electrolyte. Good water retention capacity can greatly extend the service life of the flexible Zn-based device, especially Zn air batteries that are a semi-open system in which vapor evaporation has a higher influence. Although there are already some reports about improving the water retention capacity of hydrogel electrolytes, the durability still can not satisfy commercial application. In addition, it is expected that to design a proper structure for the Zn-based batteries with hydrogel electrolytes for minimizing exposed surface to air, so as to alleviate the effect of thermal evaporation.
2. Exploring hydrogel electrolytes with high ionic conductivity. The valence state and size of the ionic radii have a significant influence on interaction between different ions and polymers, further resulting in different ionic conductivity of hydrogels. In addition, the polymer molecular weight as well as crosslinking degree also has an intimate relationship with ionic conductivity of hydrogels. The electrolytic salts may be neutral, but may also be alkaline. Therefore, it is necessary to further explore appropriate polymers and optimized preparation strategies for hydrogel electrolytes of different PH.
3. Exploring hydrogel electrolytes with novel functionalities (such as self-healing and stretchability) in allusion to various application backgrounds. Currently reported Zn-based batteries with smart electrolytes have realized fascinating functions (rubbery stretchability, self-healing ability, and good toughness). Though the performance of devices would tend to decay after repetitive usage. Therefore, the reliabilities of Zn-based devices are the urgent concern. Taking the self-healing ability as an example, some reported Zn-based batteries can self-heal without external stimulation after being broken. However, after experiencing several rounds of cutting/healing, healing efficiency would greatly decrease. Through developing a modification strategy of the polymer (such as a small fraction of permanent crosslinks), further improving strain recovery degree after stress release is still a significant topic for future research.
4. Exploring environmentally-friendly hydrogel electrolytes that are beneficial for the sustainable development of society. The urgent demands for power source devices will inevitably lead to large-scale production in the future. The development of a green synthesis process and degradable battery materials can effectively minimize environmental pollution. Although there are already some studies on biocompatible hydrogels, less attention and effort are devoted to environmentally friendly hydrogel electrolytes applied in Zn-based devices.

## Author Contributions

KL investigated literature and wrote the paper. TJ and MW conceived the idea and revised the paper. HH polished the language.

## Conflict of Interest

The authors declare that the research was conducted in the absence of any commercial or financial relationships that could be construed as a potential conflict of interest.
